# Implementable Deep Learning for Multi‐sequence Proton MRI Lung Segmentation: A Multi‐center, Multi‐vendor, and Multi‐disease Study

**DOI:** 10.1002/jmri.28643

**Published:** 2023-02-17

**Authors:** Joshua R. Astley, Alberto M. Biancardi, Paul J. C. Hughes, Helen Marshall, Guilhem J. Collier, Ho‐Fung Chan, Laura C. Saunders, Laurie J. Smith, Martin L. Brook, Roger Thompson, Sarah Rowland‐Jones, Sarah Skeoch, Stephen M. Bianchi, Matthew Q. Hatton, Najib M. Rahman, Ling‐Pei Ho, Chris E. Brightling, Louise V. Wain, Amisha Singapuri, Rachael A. Evans, Alastair J. Moss, Gerry P. McCann, Stefan Neubauer, Betty Raman, C‐MORE/PHOSP‐COVID Collaborative Group, Jim M. Wild, Bilal A. Tahir

**Affiliations:** ^1^ POLARIS, Department of Infection, Immunity & Cardiovascular Disease The University of Sheffield Sheffield UK; ^2^ Department of Oncology and Metabolism The University of Sheffield Sheffield UK; ^3^ Sheffield Teaching Hospitals NHS Foundation Trust Sheffield UK; ^4^ Royal National Hospital for Rheumatic Diseases Royal United Hospital NHS Foundation Trust Bath UK; ^5^ Arthritis Research UK Centre for Epidemiology, Division of Musculoskeletal and Dermatological Sciences, School of Biological Sciences, Faculty of Biology, Medicine and Health University of Manchester, Manchester Academic Health Sciences Centre Manchester UK; ^6^ Division of Cardiovascular Medicine, Radcliffe Department of Medicine, National Institute for Health Research (NIHR) Oxford Biomedical Research Centre (BRC) University of Oxford Oxford UK; ^7^ MRC Human Immunology Unit University of Oxford Oxford UK; ^8^ The Institute for Lung Health, NIHR Leicester Biomedical Research Centre University of Leicester Leicester UK; ^9^ Department of Health sciences University of Leicester Leicester UK; ^10^ University Hospitals of Leicester NHS Trust University of Leicester Leicester UK; ^11^ Department of Cardiovascular Sciences University of Leicester Leicester UK; ^12^ Insigneo Institute for In Silico Medicine The University of Sheffield Sheffield UK

**Keywords:** deep learning, segmentation, lung, CNN

## Abstract

**Background:**

Recently, deep learning via convolutional neural networks (CNNs) has largely superseded conventional methods for proton (^1^H)‐MRI lung segmentation. However, previous deep learning studies have utilized single‐center data and limited acquisition parameters.

**Purpose:**

Develop a generalizable CNN for lung segmentation in ^1^H‐MRI, robust to pathology, acquisition protocol, vendor, and center.

**Study type:**

Retrospective.

**Population:**

A total of 809 ^1^H‐MRI scans from 258 participants with various pulmonary pathologies (median age (range): 57 (6–85); 42% females) and 31 healthy participants (median age (range): 34 (23–76); 34% females) that were split into training (593 scans (74%); 157 participants (55%)), testing (50 scans (6%); 50 participants (17%)) and external validation (164 scans (20%); 82 participants (28%)) sets.

**Field Strength/Sequence:**

1.5‐T and 3‐T/3D spoiled‐gradient recalled and ultrashort echo‐time ^1^H‐MRI.

**Assessment:**

2D and 3D CNNs, trained on single‐center, multi‐sequence data, and the conventional spatial fuzzy c‐means (SFCM) method were compared to manually delineated expert segmentations. Each method was validated on external data originating from several centers. Dice similarity coefficient (DSC), average boundary Hausdorff distance (Average HD), and relative error (XOR) metrics to assess segmentation performance.

**Statistical Tests:**

Kruskal–Wallis tests assessed significances of differences between acquisitions in the testing set. Friedman tests with post hoc multiple comparisons assessed differences between the 2D CNN, 3D CNN, and SFCM. Bland–Altman analyses assessed agreement with manually derived lung volumes. A *P* value of <0.05 was considered statistically significant.

**Results:**

The 3D CNN significantly outperformed its 2D analog and SFCM, yielding a median (range) DSC of 0.961 (0.880–0.987), Average HD of 1.63 mm (0.65–5.45) and XOR of 0.079 (0.025–0.240) on the testing set and a DSC of 0.973 (0.866–0.987), Average HD of 1.11 mm (0.47–8.13) and XOR of 0.054 (0.026–0.255) on external validation data.

**Data Conclusion:**

The 3D CNN generated accurate ^1^H‐MRI lung segmentations on a heterogenous dataset, demonstrating robustness to disease pathology, sequence, vendor, and center.

**Evidence Level:**

4.

**Technical Efficacy:**

Stage 1.

Imaging of the lungs is a key component in the management of patients with respiratory diseases and facilitates their diagnosis, treatment planning, monitoring, and assessment. Imaging modalities such as computed tomography (CT) and proton MRI (^1^H‐MRI) enable the visualization and quantification of anatomical features within the lungs.^1^, ^2^ High‐resolution CT has traditionally represented the reference standard in clinical practice for structural lung imaging due to its impeccable resolution (~1 mm^3^) and ubiquitous availability.^3^ ^1^H‐MRI has historically been limited in the management of patients with respiratory diseases due to the low proton density and fast signal decay within the lungs, which pose inherent challenges for the modality.^4^ However, recent advances in sequence development and coil design have improved structural detail via ultrashort and zero echo‐time sequences which increase the resolution to approximately that of CT (~1.5 mm^3^), enabling the use of ^1^H‐MRI in numerous pulmonary imaging applications.^5^ Furthermore, ^1^H‐MRI uses non‐ionizing radiation and therefore can be utilized for pediatric patient care and treatment monitoring where longitudinal imaging studies are required.

Segmentation of the lungs in ^1^H‐MRI is required to delineate the lung cavity from other nearby features and has numerous applications, such as disease characterization,^6^ treatment planning^7^ and longitudinal assessment.^8^ Lung segmentation is also required for the computation of quantitative dynamic contrast‐enhanced and oxygen‐enhanced MRI, which evaluate lung perfusion and ventilation, respectively.^5^ In addition, surrogates of ventilation can be derived from non‐contrast, multi‐inflation ^1^H‐MRI, requiring the segmentation of the lung parenchyma at different volumes.^9^ Segmentation of pathological lungs, in particular, represents a challenge due to the relative similarity in signal intensity between aerated and non‐aerated lung tissue and the presence of various pathological patterns such as ground glass opacities, consolidation, and bronchiectasis.

Conventional image processing and machine learning approaches have traditionally been used for lung segmentation in ^1^H‐MRI; these include semi‐automatic thresholding, clustering and region growing methods.^1^ Spatial fuzzy c‐means (SFCM) is a clustering method that employs spatial information to modify cluster membership and has been used successfully as a semi‐automated ^1^H‐MRI lung segmentation method.^10^, ^11^ However, although these methods achieved varying degrees of success, they remain semi‐automated in nature. Time‐consuming manual correction is often required to modify semi‐automated methods based on MRI sequence or readout parameters.

In recent years, deep learning (DL) has largely superseded classical image processing, such as thresholding, and conventional machine learning, such as clustering, for medical image segmentation applications. Convolutional neural networks (CNNs) have emerged as the dominant DL approach and have been used in numerous pulmonary image segmentation applications. A recent review of DL applications in lung image segmentation indicated that studies predominantly utilized CT imaging and single‐center datasets.^12^ This leads to reduced performance when deploying DL models across multiple centers due to variations in training and testing set distributions.^13^ Due to variations in MR acquisition protocols or vendor, the large‐scale segmentation of ^1^H‐MRI represents a significant challenge for the deployment of implementable DL models. Multi‐center datasets have been used for other DL‐based lung segmentation applications such as the use of the COPDGene dataset in CT fissure detection and segmentation^14^; however, large‐scale DL investigations are yet to be conducted for ^1^H‐MRI lung segmentation. Consequently, there is a pressing need for a multi‐center implementable approach to ^1^H‐MRI segmentation that can be deployed regardless of specific MR imaging parameters or patient pathology.

In this study, we hypothesized that a generalizable DL‐based segmentation algorithm can accurately delineate the lung cavity across a multi‐center, multi‐vendor, and multi‐disease ^1^H‐MRI dataset. We aimed to develop and compare ^1^H‐MRI DL segmentation networks with a conventional segmentation approach to automatically segment the lungs on ^1^H‐MRI scans.

## Materials and Methods

### Patient Data

All studies received ethical approval from the relevant institutional review boards with participants (or their guardians) providing informed written consent. Appropriate consent and permissions have been granted by the sponsors to utilize these data for retrospective purposes. All data were anonymized, and all investigations were conducted in accordance with the appropriate guidelines and regulations.

^1^H‐MRI scans used in this study were retrospectively collected from several research imaging studies and patients referred for clinical pulmonary MRI scans. The dataset comprised 809 ^1^H‐MRI scans from 31 healthy participants with a median age (range) of 34 (23, 76); 66% males, 34% females and 258 participants with various pulmonary pathologies with a median age (range) of 57 (6, 85); 58% males, 42% females. Scans acquired at different inflation levels, longitudinal, and intrasession reproducibility scans were included in the dataset, resulting in a larger number of 3D scans than participants. A breakdown of patient data and demographics, stratified by disease, is included in Table 1.

**TABLE 1:** Summary of Patient Data

Disease	Number of Subjects	Number of Scans	Age^a^	Sex^a^
			Median (range)	Frequency (%)
Asthma	17	89	50 (15, 73)	5 M (29%), 12 F (71%)
Post‐COVID‐19	147	376	57 (21, 83)	97 M (66%), 49 F (34%)
Cystic fibrosis	26	82	18 (6, 48)	12 M (46%), 14 F (54%)
Healthy	31	103	34 (23, 76)	19 M (66%), 10 F (34%)
ILD^b^	46	83	69 (44, 83)	25 M (54%), 21 F (46%)
Investigation for possible airways disease	4	15	50 (46, 64)	0 M (0%), 4 F (100%)
Lung cancer	18	59	72 (35, 85)	11 M (61%), 7 F (39%)
Total	289	809	56 (6, 85)	168 M (59%), 117 F (41%)

^a^ Patient demographic data were unavailable for four participants.

^b^ Contains connective tissue disease‐associated interstitial lung disease (CTD‐ILD), hypersensitivity pneumonitis (HP), idiopathic pulmonary fibrosis (IPF) and drug‐induced ILD (DI‐ILD).

M = male; F = female; ILD = interstitial lung disease.

### ^1^H‐MRI Protocol

The dataset used in this study contained ^1^H‐MRI acquired with a range of sequences and readout parameters from three distinct centers in the United Kingdom. ^1^H‐MRI acquisition details are summarized in Table 2.

**TABLE 2:** ^1^H‐MRI Acquisition Details

	Acquisition 1	Acquisition 2	Acquisition 3	Acquisition 4	Acquisition 5	External Validation 1	External Validation 2
Centre	Center 1	Center 1	Center 1	Center 1	Center 1	Center 2	Center 3
Scanner	GE HDx	Philips Ingenia	GE HDx	GE HDx	GE HDx	Siemens Skyra	Siemens Prisma
Field strength	1.5 T	3 T	1.5 T	1.5 T	1.5 T	3 T	3 T
Coil	8‐channel cardiac	Body	8‐element cardiac	Body	Body	Body	Body
Sequence	UTE (kooshball)	SPGR	SPGR	SPGR	SPGR	SPGR	SPGR
Sequence dimension	3D	3D	3D	3D	3D	3D	3D
Acquisition orientation	Axial	Coronal	Coronal	Coronal	Coronal	Coronal	Coronal
Inflation level	FRC (free‐breathing gated on expiration)	INSP/EXP	RV, TLC, FRC + bag^a^	FRC + bag^a^	FRC + bag^a^	INSP/EXP	INSP/EXP
Slice thickness (mm)	~1.5	5	3 or 4	5	10	3	3
Interslice distance (mm)	~1.5	2.5	3 or 4	5	10	3	3
In‐plane resolution (mm^2^)	~1.5 × 1.5	~2 × 2	~3 × 3 or ~4 × 4	~4 × 4	~4 × 4	~3.13 × 3.13	~3.13 × 3.13
TR/TE (milliseconds)	2.8/0.078	1.9/0.6	1.8/0.7	1.9/0.6	1.9/0.6	1.9/0.7	1.9/0.7
Flip angle (°)	4	3	3	5	5	3	3
Field of view (cm)	~35–48	~38–40	~35–48	~35–48	~35–48	~40	~40
Bandwidth (kHz)	±125	±321.4	±166.6	±166.6	±166.6	±200.3	±200.3

^a^ Bag volume was titrated based on standing height and ranges from 400 mL to 1 L.

FRC = functional residual capacity; RV = residual volume; TLC = total lung capacity; INSP = inspiratory; EXP = expiratory; SPGR = spoiled‐gradient recalled echo; UTE = ultrashort echo time.

Spoiled‐gradient echo (SPGR) and ultrashort echo‐time (UTE) ^1^H‐MRI scans were collected from center 1 and originated from several research and clinical studies conducted between 2014 and 2022. The data were used for training and testing DL networks containing a total of 643 scans from 207 participants and included five distinct MR sequence and readout parameter configurations (see Table 2). These acquisitions included differences in scanner manufacturer, sequence, field strength, lung inflation level, in‐plane resolution, and slice thickness.

SPGR ^1^H‐MRI scans collected from center 2 and center 3 and originated from a single clinical study conducted between 2021 and 2022. They were used for external validation with a total of 110 scans from 55 participants (center 2) and 54 scans from 27 participants (center 3) acquired 3 to 12 months after hospitalization due to COVID‐19. Each participant underwent an inspiratory and expiratory scan, resulting in two scans per subject. Acquisition details are provided in Table 2.

### ^1^H‐MRI Segmentations

All ^1^H‐MRI scans (*n* = 809) had corresponding, manually edited segmentations, representing the lung parenchyma. These segmentations were used as ground‐truth delineations of the lung cavity volume, exclusive of major airways. Segmentations were pooled retrospectively and were originally generated manually or using a variety of semi‐automated methods.^10^, ^15^, ^16^ Subsequently, they were manually reviewed and edited by several experienced observers (B.A.T had 10 years, H.M had 7 years, G.J.C had 6 years, P.J.C.H had 5 years, A.M.B had 5 years, H.F.C had 4 years, L.J.S had 3.5 years, and J.R.A had 3 years of experience in editing lung segmentations) with each observer segmenting different cases within the dataset using the ITK‐SNAP software (ITK‐SNAP, University of Pennsylvania, PA, USA). Airways were removed down to the third generation, and care was taken to ensure that no more than two connected components were present in the segmentations, thus removing any potentially incorrect stray voxels.

### Convolutional Neural Networks

The proposed networks consisted of a 2D and 3D implementation of the UNet CNN.^17^ All networks were trained using the medical imaging DL framework NiftyNet (0.6.0)^18^ built on top of TensorFlow (1.14).^19^ To ensure an adequate comparison between the two CNNs, training was performed on an NVIDIA Tesla V100 graphical processing unit (GPU) (Nvidia Corporation, Santa Clara, CA, USA) with 16 GB of RAM for the same length of time, thereby normalizing the performance in terms of computational efficiency and resources. Each network was trained for 120 hours.

#### 2D UNet

A 2D UNet^20^ architecture was used with varying kernel sizes from 3 × 3 × 3 to 1 × 1 × 1 depending on the layer of the network. An input spatial window size of 128 × 128 × 1 and a volume padding size of 24 × 24 × 0 was implemented to maintain consistent image dimensions. Each network was trained with a partial rectified linear unit (PReLU) activation function,^21^ Adam optimization^22^ and binary cross‐entropy loss function. A learning rate of 1 × 10^−5^ and batch size of 1 were used for 123 training epochs. A decay of 1 × 10^−6^ and L2 regularization were implemented to minimize overfitting.

#### 3D UNet

A 3D implementation of the UNet, referred to as the nn‐UNet was used.^17^ Convolution operations varied in kernel size from 3 × 3 × 3 to 1 × 1 × 1 depending on the layer of the network. The network also made use of instance and batch normalization to reduce the covariate shift between network layers. An isotropic spatial window size of 96 × 96 × 96 was used. Each network was trained with a PReLU activation function,^21^ Adam optimization^22^ and binary cross‐entropy loss function. A learning rate of 1 × 10^−5^ and batch size of 2 were used for 227 training epochs. A decay of 1 × 10^−6^ and L2 regularization were selected to minimize overfitting.

#### DATA AUGMENTATION

Data augmentation was employed before 3D scans were fed into the network to increase the variability of the training images. The augmentation method did not increase the total size of the dataset but instead used random rotation and scaling factors to modify scans before entering the network. Rotation angles of −10° to 10° and scaling values of −10% to 10% were applied for each epoch, selected based on previous research investigations.^23^ Augmentation techniques were constrained to the above limits to produce physiologically plausible scans.

#### TRAINING AND TESTING SETS

Fifty scans from 50 participants, with 10 scans from each distinct acquisition in center 1, were randomly selected as a testing set. This constituted approximately 8% of the total number of scans from center 1 and 25% of the total number of participants. This was done to ensure that no participant was included concurrently in the training and testing sets and that only one scan per participant was included in the testing set. In addition, two external validation cohorts from centers 2 and 3 were used to further validate the DL frameworks. Therefore, as a proportion of the total dataset, approximately 27% and 46% of the data in terms of scans and participants were used for testing, respectively. Numbers of scans and participants in the training, testing, and external validation datasets are shown in Table 3.

**TABLE 3:** Breakdown of Training and Testing Strategy With External Validation

	Image Acquisition	Number of Scans	Number of Participants
Training	Total	593	157^a^
	Acquisition 1	89	44
	Acquisition 2	78	39
	Acquisition 3	242	65
	Acquisition 4	99	26
	Acquisition 5	85	33
Testing	Total	50	50
	Acquisition 1	10	10
	Acquisition 2	10	10
	Acquisition 3	10	10
	Acquisition 4	10	10
	Acquisition 5	10	10
External validation	Total	166	82
	External validation 1	110	55
	External validation 2	54	27

^a^ The number of unique participants in the training set. The totals for each acquisition in the training set are greater than this number as some participants have scans from multiple acquisitions.

### Conventional Approach: Spatial Fuzzy c‐Means

A conventional approach commonly used for ^1^H‐MRI segmentation, namely, SFCM, was used.^10^ Images were initially bilaterally filtered to remove noise and maintain edges.^24^ SFCM differs from generic FCM algorithms in that it assumes that voxels in close spatial proximity will have a high correlation with each other and hence have similarly high membership to the same cluster. This spatial information will modify the membership value if, for instance, the voxel is noisy yet highly spatially correlated and consequently would have been incorrectly classified. The optimal number of clusters was manually selected by A.M.B based on previous experience in the clinical translation of this technique. Traditional FCM methods assign *N* pixels to *C* clusters via fuzzy memberships yet do not make use of nearby pixels during the iteration process. By taking into account, the membership of voxels within a predefined window (5 × 5 in this work), SFCM will weigh the central voxel depending on the provided weighting variables^25^ and thus is expected to generate more accurate segmentations.^10^

### Quantitative Evaluation

Segmentations generated by DL and SFCM were compared to manually annotated segmentations and quantitatively evaluated using the following voxel‐based evaluation metrics. The overlap‐based Dice similarity coefficient (DSC) metric assesses the overlap between ground truth (*GT*) and output (*OP*) segmentations and is defined as follows^26^:

(1) $$\mathrm{DSC}=2\frac{\left|\mathrm{OP}\cap \mathrm{GT}\right|}{\left|\mathrm{OP}\right|+\left|\mathrm{GT}\right|}$$

The average boundary Hausdorff distance (Average HD) assesses the conformity of boundaries between *GT* and *OP* segmentations and is defined as follows^27^:

(2) $$\mathrm{HD}\left(\mathrm{OP},\mathrm{GT}\right)=\max(h\left(\mathrm{OP},\mathrm{GT}\right),h\left(\mathrm{GT},\mathrm{OP})\right)$$

where $h\left(\mathrm{OP},\mathrm{GT}\right)$ represents the directed Hausdorff distance between the sets of *OP* and *GT* voxels at the boundary, *op* represents an individual boundary voxel in the set OP, and *gt* represents an individual boundary voxel in GT. Further, $h\left(\mathrm{OP},\mathrm{GT}\right)$ is defined as:

(3) $$h\left(\mathrm{OP},\mathrm{GT}\right)=\max_{\mathrm{op}\in \mathrm{OP}}\min_{\mathrm{gt}\in \mathrm{GT}}\left\|\mathrm{OP}-\mathrm{GT}\right\|$$

$\text{where}\;\left\|\mathrm{OP}-\mathrm{GT}\right\|$ is the Euclidean distance between OP and GT.

The relative error metric (XOR) is an error‐based metric, which is expected to correlate with the manual editing time required to correct the *OP* segmentation^28^ and is defined as follows:

(4) $$\mathrm{XOR}=\frac{\left|\mathrm{OP}\cap {\mathrm{GT}}^{\prime }\right|+\left|{\mathrm{OP}}^{\prime }\cap \mathrm{GT}\right|}{\left|\mathrm{GT}\right|}$$

where *OP′* and *GT′* are the complements of *OP* and *GT*, respectively.

### Statistical Analysis

The normality of the data was assessed using Shapiro–Wilk tests; if normality was not satisfied, non‐parametric tests were conducted. Kruskal–Wallis tests for multiple comparisons were used to assess differences in segmentation performance between center 1 image acquisitions (see Table 2). One‐way repeated‐measures analysis of variance (ANOVA) with Tukey's test or Friedman tests with corrected Dunn's method for post hoc multiple comparisons were used to assess differences in segmentation performance between the 2D UNet, 3D UNet and SFCM methods for center 1 data. Bland–Altman analyses were conducted to compare the 2D UNet‐, 3D UNet‐ and SFCM‐generated segmentations on external validation data. ANOVA or Friedman tests were used to assess differences between segmentation methods on external validation cohorts from centers 2 and 3. Furthermore, independent *t*‐tests with Welch's correction or Mann–Whitney U tests were used to assess differences between expiratory and inspiratory segmentations in external validation data. Statistical analyses were conducted using GraphPad Prism 9.2.0 (GraphPad Software, San Diego, CA). A *P* value of <0.05 was considered statistically significant.

## Results

### Qualitative Evaluation

**FIGURE 1:**
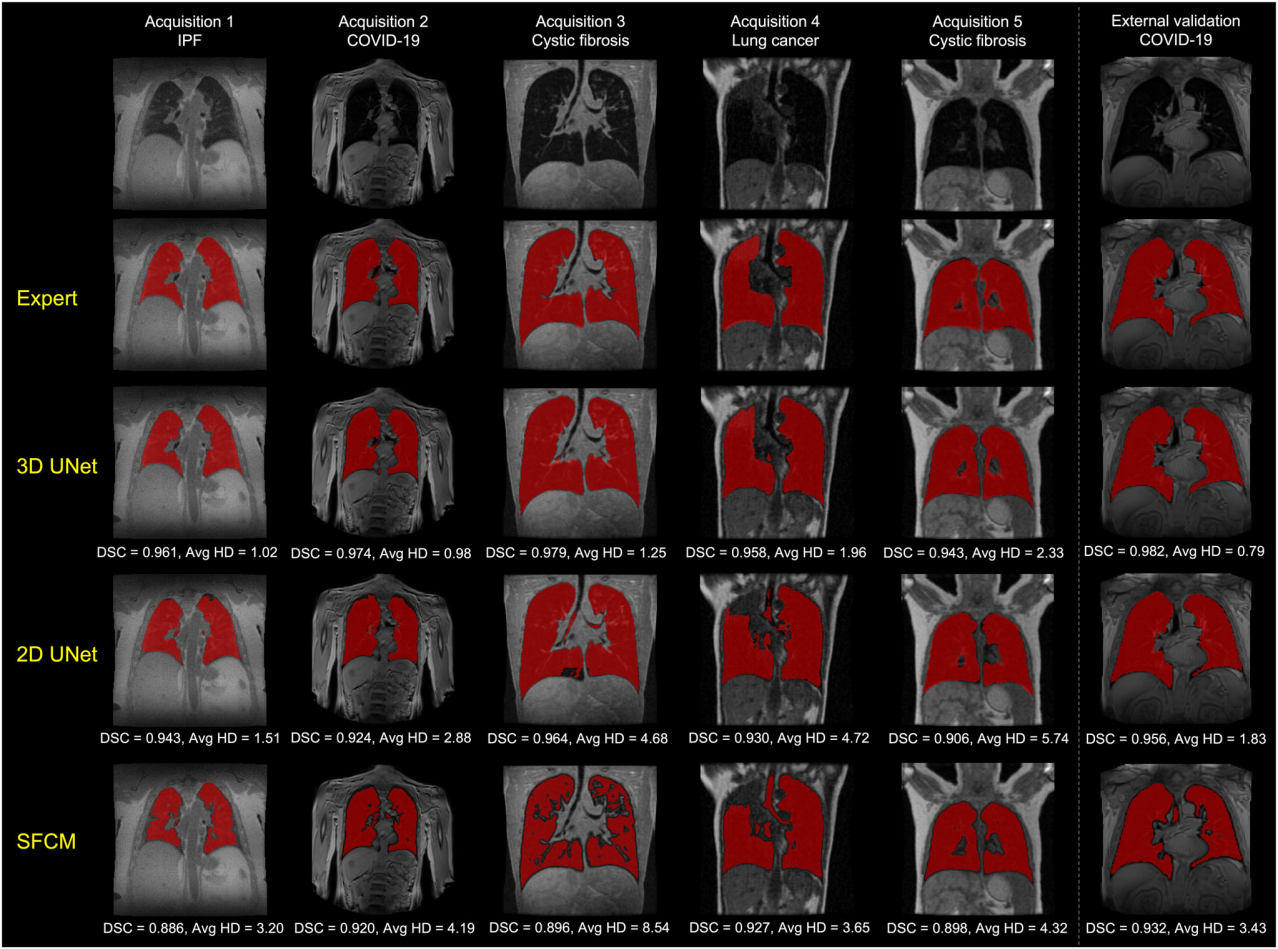
Example coronal slices showing the ^1^H‐MRI scans (row 1), the ^1^H‐MRI scans overlaid with manual segmentations (row 2) and segmentations generated by the 3D UNet, 2D UNet and spatial fuzzy c‐means (SFCM) methods (rows 3–5) for six representative cases. Dice similarity coefficient (DSC) and average Hausdorff distance (HD) values are provided for each case. Example slices were left uncropped to display differences in field of view and arm position between acquisitions.

**FIGURE 2:**
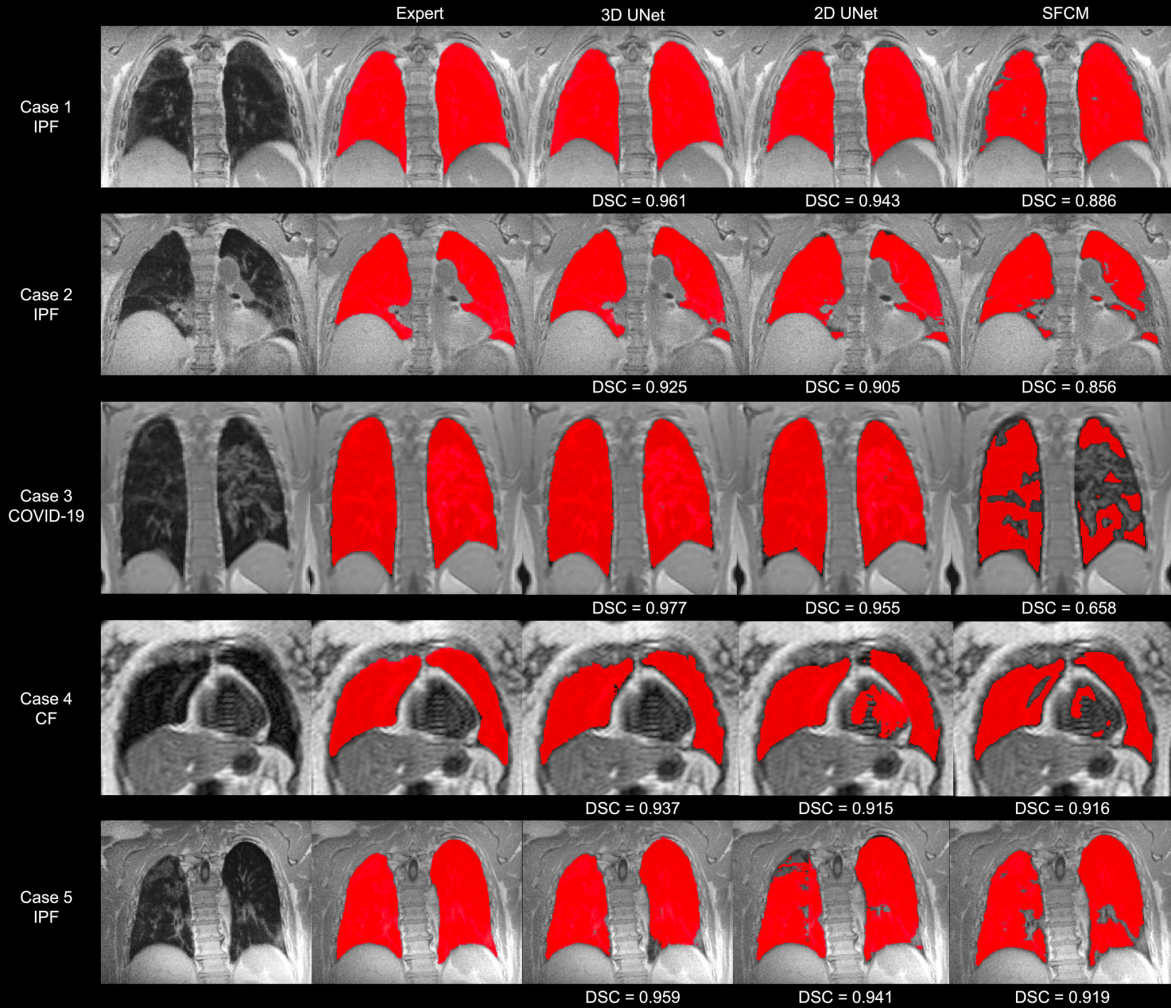
Example coronal slices showing ^1^H‐MRI scans that exhibit challenging features such as artifacts, ground glass opacities, consolidation, and bronchiectasis for five cases with corresponding expert, deep learning, and spatial fuzzy c‐means (SFCM) segmentations. Dice similarity coefficient (DSC) values are provided for each case and method.

Figure 1 shows the segmentations generated by the 2D UNet, 3D UNet and SFCM methods in comparison to the manually edited segmentations for six cases, where a range of pulmonary pathologies, centers, and MR sequences were chosen to demonstrate each method's performance. For all cases, the 3D UNet exhibited improved performance over its 2D analog and the SFCM method; this superior performance was maintained for the external validation dataset. Cases with challenging features such as artifacts, ground glass opacities, consolidation and bronchiectasis are displayed in Fig. 2 along with expert, DL and SFCM segmentations. The 3D UNet exhibited improved performance on these cases compared to the other approaches tested; however, some differences were observed with expert segmentations, particularly when areas of high signal intensity were adjacent to the border of the lung cavity.

### Center 1 Evaluation

**FIGURE 3:**
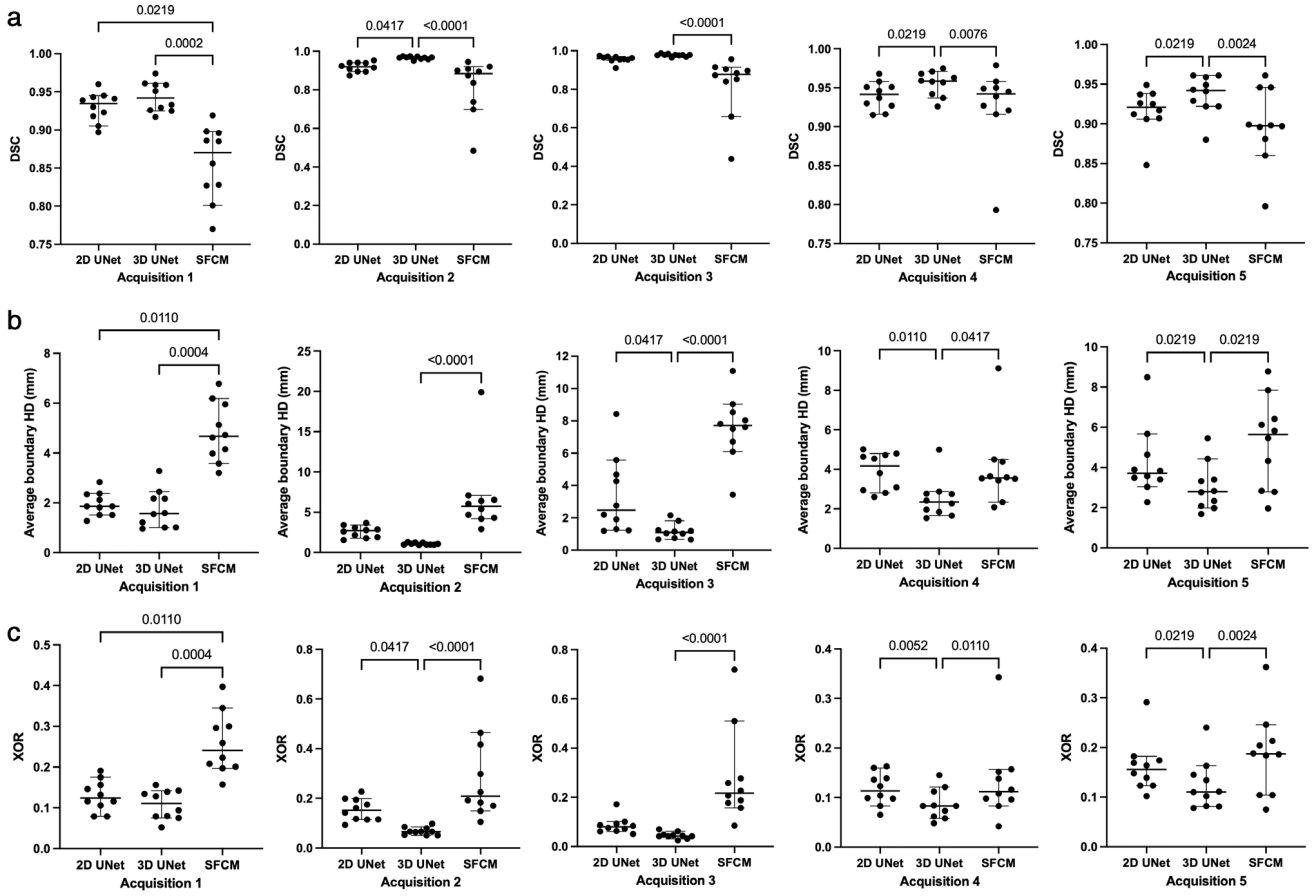
Comparison of segmentation performance for each of the methods using the (a) Dice similarity coefficient (DSC), (b) average Hausdorff distance (HD), and (c) relative error (XOR) metrics. Significances of differences between deep learning methods and spatial fuzzy c‐means (SFCM) as assessed by Friedman tests with Dunn's method are displayed for each metric.

**FIGURE 4:**
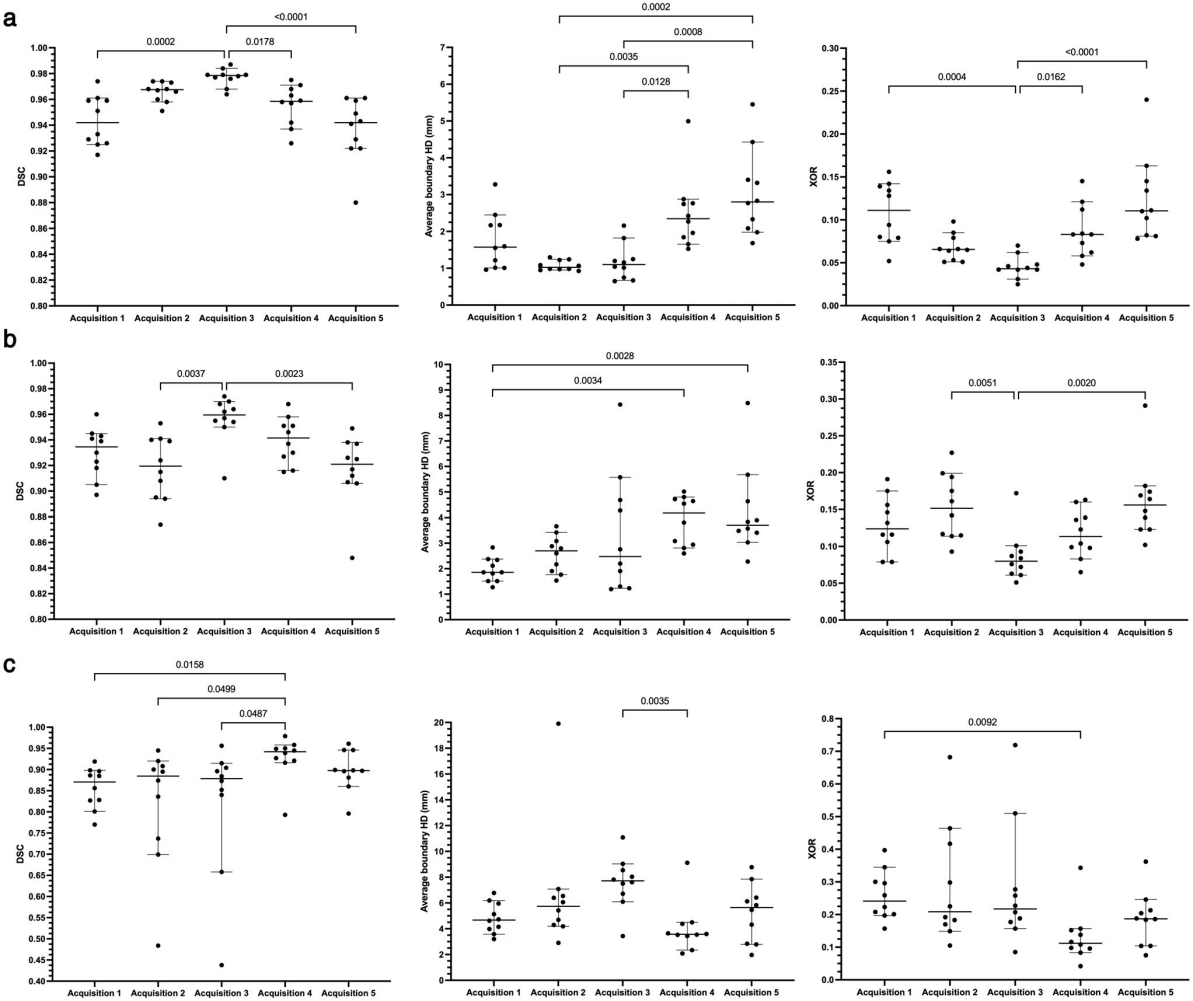
Comparison of segmentation performance across acquisition protocols for the Dice similarity coefficient (DSC), average Hausdorff distance (HD) and relative error (XOR) metrics for (a) 3D UNet, (b) 2D UNet, and (c) spatial fuzzy c‐means (SFCM) methods. Significant differences between image acquisitions as assessed by Kruskal–Wallis tests are given for each metric.

Quantitative results for the 2D UNet, 3D UNet and SFCM method are displayed in Table 4. Results demonstrated that the 3D UNet generated superior segmentations across all three metrics for each acquisition. The 3D UNet achieved a median (range) DSC, Average HD and XOR of 0.961 (0.880, 0.987), 1.63 mm (0.65, 5.45) and 0.079 (0.025, 0.240), respectively, on testing data from center 1. Both the DL‐based approaches outperformed the SFCM method across all three metrics. Network training performance and convergence for the 3D and 2D UNets are illustrated graphically in the Supplementary material S1. Our 3D UNet trained model is publicly available at https://github.com/POLARIS-Sheffield/1H-MRI-segmentation. In Fig. 3, performance between segmentation methods is shown per MR acquisition configuration for all metrics. The 3D UNet significantly outperformed the SFCM method in all comparisons and the 2D UNet in almost all comparisons. The 2D UNet statistically outperformed the SFCM on acquisition 1 data only. Figure 4 displays graphically the performance of the (a) 3D UNet, (b) 2D UNet and (c) SFCM methods for each metric. All methods exhibited statistically significant differences between some of the acquisitions; however, the 3D UNet exhibited the smallest range between least and best performing MR acquisition. The 3D UNet produced the most accurate segmentations for a single acquisition (acquisition 3) when using all three metrics; in contrast, the 2D UNet and SFCM methods did not consistently exhibit superior performance for a specific acquisition across metrics.

**TABLE 4:** Quantitative Results for the Testing Set (*n* = 50), External Validation 1 (*n* = 110), and External Validation 2 (*n* = 54) Using the DSC, Average HD (mm), and XOR metrics for the SFCM, 2D UNet, and 3D UNet methods

Acquisition	SFCM			2D UNet			3D UNet		
	DSC	Average HD (mm)	XOR	DSC	Average HD (mm)	XOR	DSC	Average HD (mm)	XOR
	Median (range)	Median (range)	Median (range)	Median (range)	Median (range)	Median (range)	Median (range)	Median (range)	Median (range)
Acquisition 1	0.871 (0.770, 0.919)	4.67 (3.20, 6.78)	0.241 (0.157, 0.397)	0.935 (0.897, 0.960)	1.86 (1.27, 2.83)	0.124 (0.079, 0.191)	0.942 (0.917, 0.974)	1.57 (0.96, 3.28)	0.111 (0.052, 0.156)
Acquisition 2	0.885 (0.484, 0.945)	5.74 (2.90, 19.9)	0.209 (0.105, 0.682)	0.920 (0.874, 0.953)	2.70 (1.54, 3.66)	0.152 (0.093, 0.227)	0.968 (0.951, 0.974)	1.03 (0.93, 1.30)	0.065 (0.051, 0.098)
Acquisition 3	0.879 (0.438, 0.956)	7.71 (3.43, 11.1)	0.217 (0.085, 0.719)	0.960 (0.910, 0.974)	2.48 (1.20, 8.43)	0.080 (0.051, 0.172)	0.979 (0.964, 0.987)	1.10 (0.65, 2.16)	0.043 (0.025, 0.070)
Acquisition 4	0.942 (0.793, 0.979)	3.57 (2.08, 9.12)	0.112 (0.042, 0.343)	0.942 (0.915, 0.968)	4.17 (2.60, 5.01)	0.114 (0.065, 0.163)	0.959 (0.926, 0.975)	2.35 (1.53, 4.99)	0.083 (0.048, 0.145)
Acquisition 5	0.898 (0.796, 0.961)	5.64 (1.96, 8.78)	0.187 (0.075, 0.362)	0.921 (0.848, 0.949)	3.71 (2.28, 8.49)	0.156 (0.102, 0.291)	0.942 (0.880, 0.961)	2.80 (1.68, 5.45)	0.111 (0.078, 0.240)
Testing total	0.896 (0.438, 0.979)	5.28 (1.96, 19.9)	0.195 (0.042, 0.719)	0.938 (0.848, 0.974)	2.86 (1.20, 8.49)	0.123 (0.051, 0.291)	0.961 (0.880, 0.987)	1.63 (0.65, 5.45)	0.079 (0.025, 0.240)
External validation 1	0.831 (0.295, 0.949)	5.07 (2.82, 54.1)	0.290 (0.097, 0.918)	0.894 (0.477, 0.959)	4.58 (1.64, 16.7)	0.197 (0.080, 0.688)	0.973 (0.866, 0.986)	1.19 (0.53, 8.13)	0.054 (0.028, 0.255)
External validation 2	0.808 (0.170, 0.925)	5.88 (3.35, 71.9)	0.324 (0.141, 0.907)	0.902 (0.272, 0.954)	3.47 (1.79, 44.8)	0.185 (0.090, 0.912)	0.972 (0.914, 0.987)	0.96 (0.47, 3.86)	0.056 (0.026, 0.159)
External validation total	0.819 (0.170, 0.949)	5.36 (2.82, 71.9)	0.307 (0.097, 0.918)	0.894 (0.272, 0.959)	4.08 (1.64, 44.8)	0.197 (0.080, 0.912)	0.973 (0.866, 0.987)	1.11 (0.47, 8.13)	0.054 (0.026, 0.255)

Median (range) values are provided for each acquisition protocol, the combined testing set, and the external validation sets.

SFCM = spatial fuzzy c‐means; DSC = Dice similarity coefficient; Average HD = average boundary Hausdorff distance; XOR = relative error metric.

### External Data Evaluation

**FIGURE 5:**
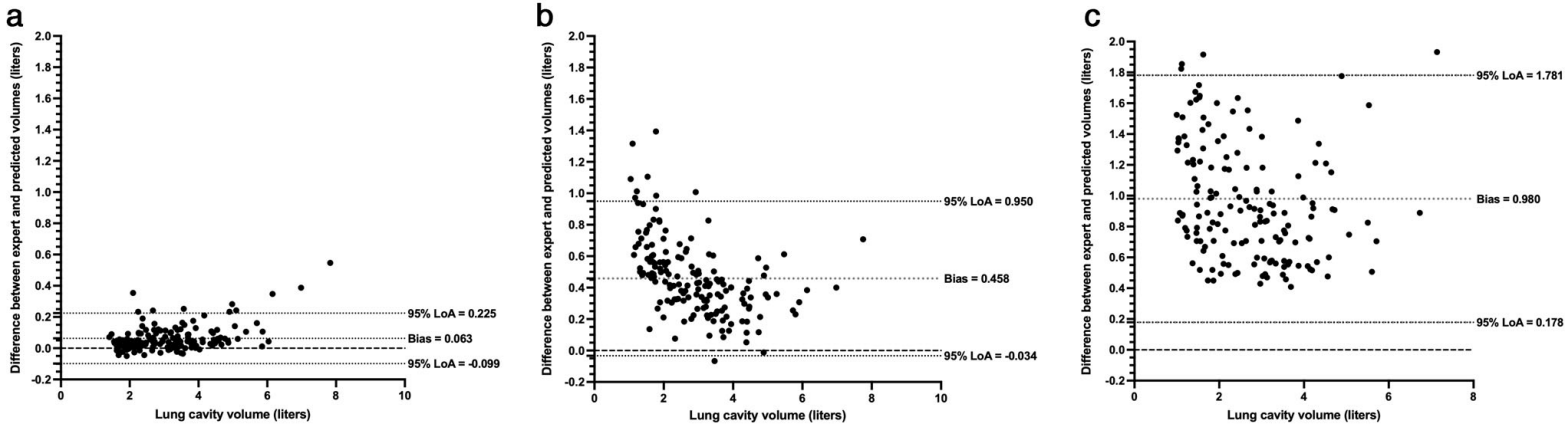
Bland–Altman agreement analysis of lung volumes for 164 external validation set cases compared to volumes derived from manual segmentations for (a) 3D UNet (b) 2D UNet, and (c) spatial fuzzy c‐means (SFCM) methods.

As shown in Table 4, improved performance over center 1 testing data was exhibited on the external validation cohorts, achieving a median (range) DSC, Average HD and XOR of 0.973 (0.866, 0.987), 1.11 mm (0.47, 8.13) and 0.054 (0.026, 0.255), respectively. The 3D UNet significantly outperformed the 2D UNet and SFCM for all three metrics across 164 external validation scans using the DSC, Average HD, and XOR metrics; distribution and comparison of segmentation performance are displayed in the Supplementary material S1. Figure 5 shows Bland–Altman analyses comparing the lung parenchymal volume of DL methods and SFCM to manually derived lung volumes for the 164 external validation scans from centers 2 and 3. The 3D UNet exhibited a significantly reduced bias compared to other methods tested and achieved a bias of 0.063 liters with limits of agreement (LoA) −0.099 to 0.225 liters.

**FIGURE 6:**
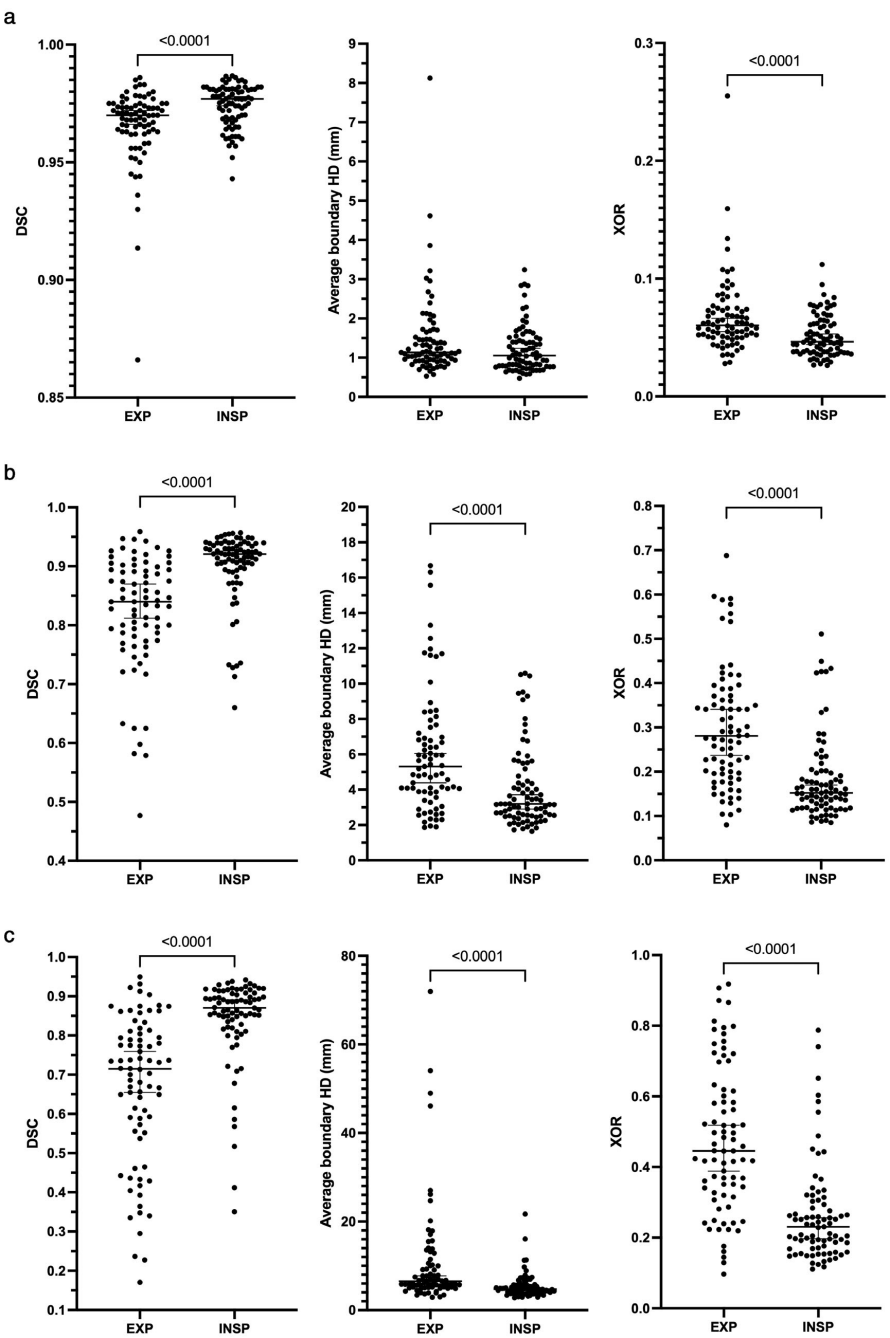
Comparison of the combined external validation datasets stratified by inspiratory and expiratory scans using the Dice similarity coefficient (DSC), average Hausdorff distance (HD) and relative error (XOR) metrics for (a) 3D UNet, (b) 2D UNet, and (c) spatial fuzzy c‐means (SFCM) methods. *P* values between inspiratory and expiratory scans are shown.

Figure 6 displays a comparison of segmentation performance between expiratory and inspiratory scans in data from centers 2 and 3 for all metrics used. For the 2D UNet and the SFCM methods, inspiratory scans were segmented more accurately than expiratory scans for all metrics. This was replicated for the 3D UNet using the DSC and XOR metrics; however, no difference was observed between inspiratory and expiratory scans using the Average HD metric (*P* = 0.06).

## Discussion

In this study, the proposed implementable DL segmentation algorithm produced accurate lung segmentations on a large, multi‐center, multi‐acquisition, multi‐disease ^1^H‐MRI dataset. Our proposed 3D CNN significantly outperformed a 2D CNN and a conventional machine learning segmentation method. In addition, it was validated on external data from two centers, acquired on different vendor scanners, demonstrating minimal bias compared to manually edited lung volumes. Differences in lung segmentation performance were observed between scans acquired at inspiratory and expiratory inflation levels.

The dataset used is diverse in terms of pulmonary pathology, center in which the scans were acquired, and image acquisition parameters, including sequence, field strength and vendor. This results in a segmentation network that is invariant to the specifics of the ^1^H‐MRI scans analyzed, relying on relevant anatomical features present in ^1^H‐MRI scans to generate segmentations. These anatomical features remain consistent regardless of acquisition parameters in contrast to other features that varied between acquisitions, such as noise patterns, arm position, or location of the lungs within the scan. CT lung segmentation methods have adopted the large, multi‐center COPDGene dataset for validation of DL segmentation models to increase generalizability.^14^ In this work, we used a large multi‐center, multi‐vendor ^1^H‐MRI dataset to demonstrate the generalizability of the DL model, allowing it to potentially be deployed across numerous centers; this could have a large impact on the pulmonary MRI field.

Furthermore, our proposed 3D UNet demonstrated high‐quality segmentations across a range of pulmonary pathologies. This exemplary performance largely extends to particularly challenging cases such as participants with idiopathic pulmonary fibrosis. Fibrotic lungs contain an increased presence of challenging pathologies, such as ground glass opacities and honeycombing, which lead to increased heterogeneity within the lung parenchyma and consequently represent challenging cases for segmentation algorithms.^29^ Similarly, ^1^H‐MRI scans from participants who were previously hospitalized for COVID‐19 can exhibit consolidation and reticulation patterns that reduce the difference in signal intensity between lung and non‐lung tissue,^30^ which our proposed model adequately accounts for.

Quantitative results and statistical tests indicated that, for all acquisitions, across all metrics, the 3D UNet significantly outperformed the SFCM method. For the majority of acquisitions and metrics, the 3D UNet significantly outperformed its 2D analog. When tested on external validation data, some degree of overfitting was present in the 2D UNet exemplified by a reduction in performance compared to testing set data from center 1; this behavior was not exhibited by the 3D UNet. Differences in performance between the 2D and 3D UNets are potentially due to the volumetric nature of the ^1^H‐MRI scans, which were acquired using 3D sequences. In addition, anatomical features primarily occur across multiple slices and thus a 3D approach to segmentation may better encapsulate these features. Comparison between DL networks was limited due to the differences in batch size and spatial windowing between the two CNNs as a result of differing memory constraints. It is possible that these differences may impact network comparisons; however, computational resources remained consistent between 2D and 3D CNNs and therefore the computational efficiency of the networks was assessed alongside segmentation performance.

Several investigators have leveraged CNNs for pulmonary MRI segmentation. For example, Zha et al used a 2D UNet to segment the lung cavity on UTE ^1^H‐MRI scans, achieving a mean DSC of 0.96 across both lungs. However, the generalizability of this method was not demonstrated due to the small dataset of the study, which only contained 45 UTE ^1^H‐MRI scans from a limited number of diseases.^31^ Tustison et al evaluated a 3D UNet CNN for isotropic ^1^H‐MRI lung cavity segmentation, achieving a mean DSC of 0.94 on a dataset of 268 scans.^32^ These studies employed a limited range of image acquisition parameters with ^1^H‐MRI scans acquired using the same scanner and from a single center. Our 3D UNet proposed here demonstrated improved performance over previous research studies on a significantly larger dataset containing scans from multiple centers with varying sequences and readout parameters. Previous works in the field of ^1^H‐MRI lung segmentation have employed either 2D^31^ or 3D^32^ approaches; here, we directly compared differences in segmentation performance between 2D and 3D segmentation networks.

Our analysis of external validation data from centers 2 and 3 indicated that all lung cavity segmentation methods show significantly reduced performance on scans acquired at expiration. This effect was less prevalent in segmentations generated by the 3D UNet where no significant difference between inflation levels was observed using the Average HD metric. Differences in performance between inflation levels may be due to the reduced contrast between the lung parenchyma and other tissues as air is expelled from the lungs and the increased heterogeneity of signal within the parenchyma caused by pathophysiological air trapping at expiration observed in some patients. In addition, segmentations of exhaled lungs have a smaller volume than those of inhaled lungs; this can potentially bias quantitative results when using voxel‐based evaluation metrics.^33^

Accurate lung segmentation of ^1^H‐MRI plays an important role in the treatment planning, monitoring, and assessment of patients with respiratory diseases as well as other applications that require the delineation of the lung cavity such as dynamic contrast‐enhanced perfusion MRI.^5^ The ability to rapidly produce lung cavity segmentations can greatly reduce cumbersome manual editing, leading to a more streamlined lung imaging workflow and thus higher clinical throughput, increasing clinical translation.

### Limitations

The ratios of MRI acquisitions present in the training set leads to potential biases toward some MR sequences or acquisitions; those with a larger number of scans may lead to improved segmentation performance for these acquisitions by the network. In particular, this study presented more acquisition 3 scans than any other acquisition in the training set, potentially leading to the increased DSC values exhibited by the 2D and 3D UNets for this acquisition. However, using the Average HD metric, no relationship between the number of scans in the training set and reduced segmentation performance can be established, indicating that these biases are minimal. This is further reinforced by the superior performance on external validation datasets demonstrated by the 3D UNet, despite the CNN never being exposed to ^1^H‐MRI scans from these centers or vendors during training. However, external validation data contained only one pulmonary pathology, namely, patients previously hospitalized with COVID‐19.

The expert segmentations used in this work delineate only the lung parenchyma inclusive of vessels and no other relevant structures, such as the airways. Various applications require the delineation of only the lung parenchyma, including the computation of clinically relevant metrics such as the ventilation defect percentage^15^ and as a precursor step to image registration of multi‐inflation proton MRI for the generation of ^1^H‐MRI surrogates of ventilation.^34^ However, in certain respiratory disorders such as obstructive sleep apnea, the segmentation of the airways is highly relevant for studying the anatomical structure of the upper airways.^35^ Future investigations may aim to integrate a multi‐label DL solution, which can segment both the lung parenchyma and airways simultaneously.

The number of MRI sequences contained within the dataset were limited. The dataset contained SPGR and UTE sequence scans i.e. proton density or T1‐weighted scans only. In addition, UTE scans were acquired with a kooshball acquisition and, therefore, other possible acquisitions, such as Floret and spiral, were not assessed. Likewise, only 3D acquisition sequences were contained in the dataset, thereby limiting its implementation to 3D sequences. The inclusion of other MRI sequences, such as steady‐state free‐precession or fast spin echo sequences, in combination with 2D and 3D MRI sequences will help to further generalize the work. In future investigations, we will aim to further validate the model with data from an increased number of centers and from MRI sequences not previously investigated.

In this work, ^1^H‐MRI lung segmentations were primarily evaluated using voxel‐wise evaluation metrics, such as the DSC. These metrics are susceptible to reduced sensitivity in segmentation evaluation as the volume of the segmentation is increased.^36^ Hence, comparisons between lung inflation levels evaluated using voxel‐based metrics are challenging. In future work, transfer learning could be employed to boost the performance on expiratory scans or more advanced data augmentation methods could be used to increase the number of expiratory scans in the training set. Similarly, comparisons between acquisitions were limited in this study because of variations in voxel resolution, resulting in large differences in the overall number of voxels between acquisitions. While the volume of the lung cavity remained largely consistent between acquisitions, the number of voxels did not; therefore, biases were introduced when using voxel‐based evaluation metrics. The subject of appropriate evaluation metrics remains lively within the medical image analysis field with recent works aiming to quantify the benefits and drawbacks of each metric.^33^ With this in mind, in this work, we employed a range of evaluation metrics; the overlap‐based DSC,^26^ the distance‐based Average HD,^27^ and the error‐based XOR metric,^28^ which each assessed a different component of segmentation accuracy. In addition, analysis of the lung cavity volume was also undertaken when evaluating external validation data as a non‐voxel‐based evaluation metric to further diversify segmentation performance evaluation.

## Conclusion

The DL‐based implementable ^1^H‐MRI segmentation network produced accurate lung segmentations across a range of pathologies, acquisitions, vendors, and centers, which could potentially have numerous applications for pulmonary MRI quantification. A 3D CNN significantly outperformed its 2D analog and a conventional segmentation method.

## Supporting information


**Data S1** Supporting Information

Supporting Information

Supporting Information
