# Supplementary material for: Implementable Deep Learning for Multi‐sequence Proton MRI Lung Segmentation: A Multi‐center, Multi‐vendor, and Multi‐disease Study
Source: J Magn Reson Imaging. 2023 Feb 17;58(4):1030–44. doi: 10.1002/jmri.28643 (PMC10946727; doi:10.1002/jmri.28643)
Supplement: Supplementary file 1 — Data S1 Supporting Information [file JMRI-58-1030-s002.docx]

**Supplementary material 1**

***Training performance and convergence***


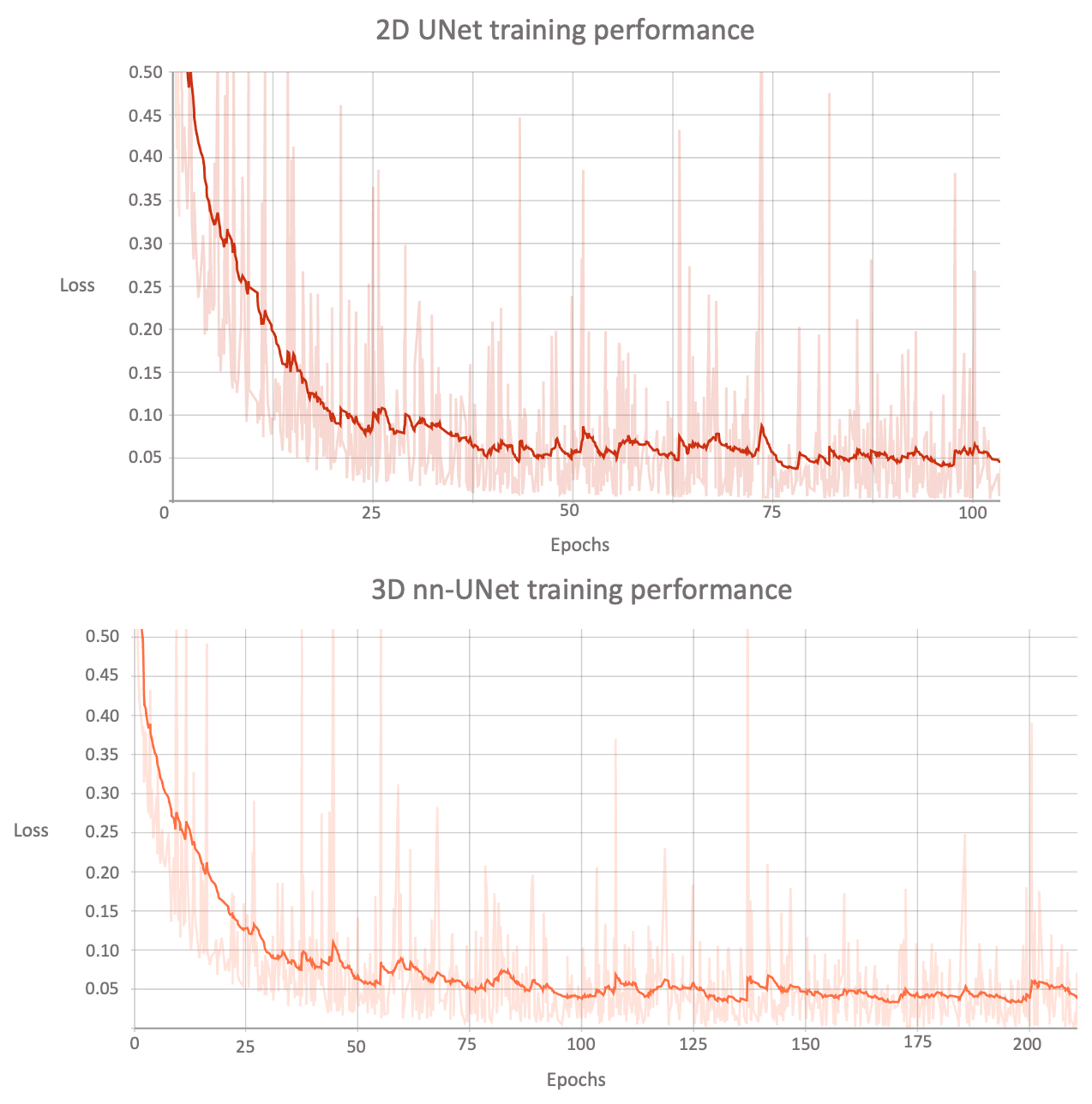


Figure S1. (Top) 2D UNet and (bottom) 3D nn-UNet training performance and convergence.

**Supplementary material 2**

***Comparison of performance on external validation data***

***
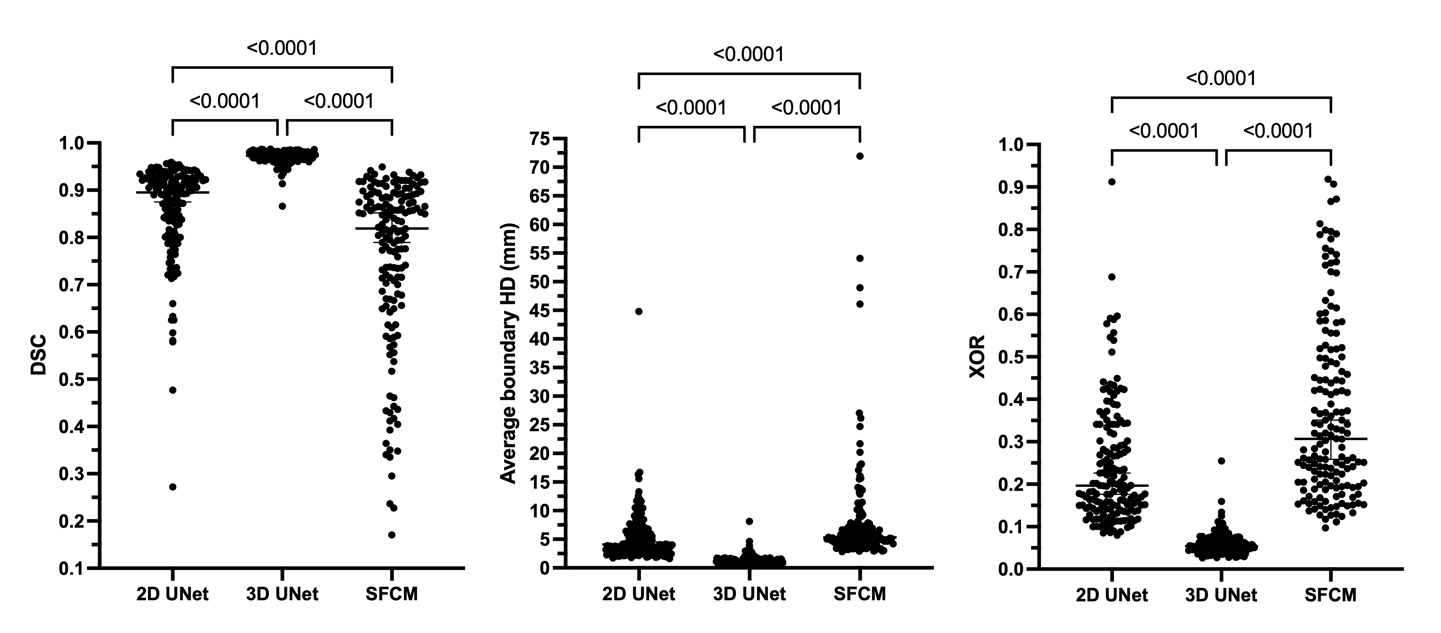
***

Figure S2. Comparison of segmentation performance on the combined external validation datasets for each of the methods using the (left) Dice similarity coefficient (DSC), (center) average boundary Hausdorff (HD) and (right) relative error (XOR) metrics. Significances of differences between deep learning methods and spatial fuzzy c-means (SFCM) as assessed by Friedman tests with Dunn’s method for multiple comparisons are displayed for each metric.
